# Changes in the management and clinical outcomes of critically ill patients without COVID-19 during the pandemic

**DOI:** 10.5935/0103-507X.20210006

**Published:** 2021

**Authors:** Iván Alfredo Huespe, Agustina Marco, Eduardo Prado, Indalecio Carboni Bisso, Pablo Coria, Nicolas Gemelli, Eduardo San Román, Marcos José Las Heras

**Affiliations:** 1 Intensive Care Unit, Hospital Italiano de Buenos Aires - Buenos Aires, Argentina.; 2 Instituto de Medicina Traslacional e Ingeniería Biomédica, Hospital Italiano de Buenos Aires, Instituto Universitario Hospital Italiano, Consejo Nacional de Investigaciones Científicas y Técnicas - Buenos Aires, Argentina.

**Keywords:** Critical care, COVID-19, SARS-CoV-2, Noninvasive ventilation, Respiratory insufficiency, Coronavirus infections, Pandemics, Cuidados críticos, COVID-19, SARS-CoV-2, Ventilação não invasiva, Insuficiência respiratória, Infecções por coronavírus, Pandemias

## Abstract

**Objective:**

To analyze whether changes in medical care due to the application of COVID-19 protocols affected clinical outcomes in patients without COVID-19 during the pandemic.

**Methods:**

This was a retrospective, observational cohort study carried out in a thirty-eight-bed surgical and medical intensive care unit of a high complexity private hospital. Patients with respiratory failure admitted to the intensive care unit during March and April 2020 and the same months in 2019 were selected. We compared interventions and outcomes of patients without COVID-19 during the pandemic with patients admitted in 2019. The main variables analyzed were intensive care unit respiratory management, number of chest tomography scans and bronchoalveolar lavages, intensive care unit complications, and status at hospital discharge.

**Results:**

In 2020, a significant reduction in the use of a high-flow nasal cannula was observed: 14 (42%) in 2019 compared to 1 (3%) in 2020. Additionally, in 2020, a significant increase was observed in the number of patients under mechanical ventilation admitted to the intensive care unit from the emergency department, 23 (69%) compared to 11 (31%) in 2019. Nevertheless, the number of patients with mechanical ventilation after 5 days of admission was similar in both years: 24 (69%) in 2019 and 26 (79%) in 2020.

**Conclusion:**

Intensive care unit protocols based on international recommendations for the COVID-19 pandemic have produced a change in non-COVID-19 patient management. We observed a reduction in the use of a high-flow nasal cannula and an increased number of tracheal intubations in the emergency department. However, no changes in the percentage of intubated patients in the intensive care unit, the number of mechanical ventilation days or the length of stay in intensive care unit.

## Abstract (pt)

**Objetivo:**

Analisar se as modificações na atenção médica em razão da aplicação dos protocolos para COVID-19 afetaram os desfechos clínicos de pacientes sem a doença durante a pandemia.

**Métodos:**

Este foi um estudo observacional de coorte retrospectiva conduzido em uma unidade de terapia intensiva clínica e cirúrgica com 38 leitos, localizada em hospital privado de alta complexidade na cidade de Buenos Aires, Argentina, e envolveu os pacientes com insuficiência respiratória admitidos à unidade de terapia intensiva no período compreendido entre março e abril de 2020 em comparação com o mesmo período no ano de 2019. Compararam-se as intervenções e os desfechos dos pacientes sem COVID-19 tratados durante a pandemia em 2020 e os pacientes admitidos em 2019. As principais variáveis avaliadas foram os cuidados respiratórios na unidade de terapia intensiva, o número de exames de tomografia computadorizada do tórax e lavados broncoalveolares, complicações na unidade de terapia intensiva e condições quando da alta hospitalar.

**Resultados:**

Observou-se, em 2020, uma redução significante do uso de cânula nasal de alto fluxo: 14 (42%), em 2019, em comparação com 1 (3%), em 2020. Além disso, em 2020, observou-se aumento significante no número de pacientes sob ventilação mecânica admitidos à unidade de terapia intensiva a partir do pronto-socorro, de 23 (69%) em comparação com 11 (31%) em 2019. Contudo, o número de pacientes com ventilação mecânica 5 dias após a admissão foi semelhante em ambos os anos: 24 (69%), em 2019, e 26 (79%) em 2020.

**Conclusão:**

Os protocolos para unidades de terapia intensiva com base em recomendações internacionais para a pandemia de COVID-19 modificaram o manejo de pacientes sem COVID-19. Observamos redução do uso da cânula nasal de alto fluxo e aumento no número de intubações traqueais no pronto-socorro. Entretanto, não se identificaram alterações na percentagem de pacientes intubados na unidade de terapia intensiva, número de dias sob ventilação mecânica ou número de dias na unidade de terapia intensiva.

## INTRODUCTION

Coronavirus disease 2019 (COVID-19) caused by severe acute respiratory syndrome coronavirus 2 (SARS-CoV-2) has rapidly developed into a global pandemic.^(1)^ The virus reproduction number (R0) is considerably higher than that of other respiratory viruses, such as influenza. Although most SARS-CoV-2-infected patients do not need hospitalization, 5% are estimated to require admission to the ICU and 2.3% would require mechanical ventilation (MV).

The aforementioned factors make COVID-19 a serious and certainly more contagious disease than influenza. Its outbreak represents a significant threat to health systems.^(2,3)^ In this context, ICUs are challenged simultaneously in terms of limited resources, infection control, protection of healthcare personnel, and adaptation to a rapidly evolving pandemic scenario.^(1)^

In Argentina, the first case was confirmed on March 3rd, 64 days after the first case was reported globally. Since then, the number of cases has increased steadily, and epidemiological controls have been intensified progressively. On March 19th, 16 days after the first reported case, the Government of Argentina announced an emergency decree establishing compulsory quarantine on a national level.^(4)^

The *Hospital Italiano de Buenos Aires* is a high complexity hospital located in the capital city of Argentina, and the first patient with COVID-19 was admitted on March 12th. An intensive care unit (ICU) protocol based on international recommendations for the COVID-19 pandemic was applied,^(5)^ including the following measures: designation of geographically isolated ICU areas, restriction of family visits, and avoidance of diagnostic and therapeutic procedures that may produce virus aerosolization, such as fibrobronchoscopy, noninvasive ventilation (NIV) and high-flow nasal cannula (HFNC).^(6-8)^ The protocol was applied to all patients admitted to the ICU with respiratory failure until reverse transcriptase-polymerase chain reaction (RT-PCR) confirmed or excluded COVID-19. However, during March and April 2020, the time to COVID-19 confirmation by RT-PCR had a 24-hour lag time since the performance of the test was centralized in a single center designated by the national government. Therefore, the pandemic situation led to a change in the standard care of ICU patients, regardless of the presence of SARS-CoV-2 infection.

The management of all ICU patients, including patients without COVID-19, has changed due to the application of COVID-19 pandemic protocols. Therefore, the aim of this study was to compare the management and outcomes of non-COVID-19 patients with respiratory failure hospitalized during the COVID-19 pandemic with similar patients hospitalized according to prepandemic protocols in 2019. Additionally, this study aims to evaluate the healthcare professionals’ perception of the impact of this new bundle of measures on patient care and prognosis.

## METHODS

A retrospective single-center cohort study was conducted in a 38-bed mixed ICU of a high complexity university hospital in Buenos Aires, Argentina. Records of all patients older than 18 years admitted to the ICU for respiratory failure during the months of March and April 2019 and from patients admitted during the same months of 2020 with a negative RT-PCR test for SARS-CoV-2 were studied. When a patient had two admissions during the same period under study, only the first admission to the ICU was considered. Patients who had pre-established do not intubate orders were excluded. This study was approved by the Research Ethics Committee of the *Hospital Italiano de Buenos Aires*.

The *Hospital Italiano* ICU multidisciplinary team is composed by intensivists, residents, nurses, respiratory therapists, dietitians, physical therapists and clinical pharmacists. No changes in the staff occurred during the two periods under study. The physician-patient ratio was 1:8, and the nurse-patient ratio was 1:3. Rounds were conducted by medical staff at least once daily, and respiratory therapists were responsible for the management of all patients on MV.

The following detailed records were included in the study: patient identification number, age, sex, comorbidities, cause of respiratory failure, Acute Physiology and Chronic Health Evaluation II (APACHE II) score and Sequential Organ Failure Assessment (SOFA) score at ICU admission, ICU respiratory management (NIV, HFNC, nonrebreather face mask or tracheal intubation), number of chest tomography (CT) scans, bronchoalveolar lavages (BAL) and changes in antibiotic therapy due to the BAL results, ICU complications (delirium measured by the Confusion Assessment Method for the Intensive Care Unit (CAM-ICU),^(9)^ ventilator-associated pneumonia diagnosis by the National Healthcare Safety Network criteria^(10)^ and pressure ulcers), and status at hospital discharge.

### Questionnaire

A questionnaire to explore the opinions of the medical staff and respiratory therapists was administered. The questions addressed the perception of the health care team regarding the management of ICU patients with respiratory failure without COVID-19 since the application of COVID-19 protocols. The survey requested the opinions of the medical staff about changes in the utilization of NIV or HFNC, indication for BAL and CT scan.

### Statistical analysis

Data that were normally distributed are presented as the means ± standard deviations, and comparisons between two groups were carried out with a two-sample t-test. Data that were not normally distributed are presented as the medians and interquartile ranges, and comparisons between groups were performed using the Mann-Whitney U test.

Categorical variables are summarized as frequencies and percentages, and comparisons were completed with the Chi-squared test or Fisher’s exact test. No imputation was made for missing data, and RStudio developed by R-Tools Technology Inc. was used for all analyses.

## RESULTS

During both periods under study, 68 patients were admitted to the ICU with respiratory failure, 35 from March to April 2019, and 33 during the same months of 2020. The median age was 68 years (interquartile range - IQR 58 - 79 years) in 2019 and 70 years old (IQR 62 - 79 years) in 2020. Groups were matched for age, body mass index, APACHE II and SOFA scores, cause of respiratory failure, and number of comorbidities. Although 66% (23 patients) of patients were male in the 2019 group, only 39% (13 patients) were male in the 2020 group. The single significant difference between the two datasets was the percentage of tobacco users, which was higher in 2020 (18%) than in 2019 (3%). All patient characteristics are portrayed in table 1.

**Table 1:** Demographic and clinical characteristics at baseline

Variables	All	2019	2020	p value
	(n = 68)	(n = 35)	(n = 33)	
Age (years)	70 (59 - 79)	68 (58 - 79)	70 (62 - 79)	0.8
Sex male	36 (52)	23 (66)	13 (39)	0.05
Body mass index (kg/m^2^)	24.3 (21.7 - 29.5)	24.3 (22 - 28.69)	24.3 (21.3 - 29.9)	0.57
APACHE II score	19 (12 - 23)	19 (11 - 25)	18 (15 - 22)	0.88
SOFA score	5.5 (3.0 - 9.0)	6.5 (3.0 - 9.0)	5.5 (4.0 - 9.0)	0.68
Causes of respiratory failure				
COPD exacerbation	9 (13)	5 (14)	4 (12)	0.99
Community-acquired pneumonia	18 (26)	11 (31)	7 (21)	0.42
Hospital-acquired pneumonia	6 (8)	5 (14)	1 (3)	0.2
Nonpulmonary septic shock	12 (18)	4 (11)	8 (24)	0.35
Congestive heart failure	10 (14)	4 (11)	6 (18)	0.5
Others	13 (19)	6 (17)	7 (21)	0.76
Number of underlying comorbidities				
No comorbidities	31 (46)	18 (51)	13 (40)	0.34
One comorbidity	23 (34)	11 (32)	12 (36)	0.79
Two or more	14 (21)	5 (17)	8 (24)	0.55
Underlying comorbidity				
COPD	14 (21)	6 (17)	8 (24)	0.77
Hypertension	33 (49)	16 (46)	17 (51)	0.81
Asthma	2 (3)	1 (3)	1 (3)	0.99
Smoking history	7 (10)	1 (3)	6 (18)	0.04*
Diabetes	9 (13)	6 (17)	3 (9)	0.31
Immunosuppression	23 (34)	12 (34)	11 (33)	0.99
Chronic renal disease	13 (19)	4 (11)	9 (27)	0.22
Congestive heart failure	14 (21)	8 (22)	6 (18)	0.77
Obesity	9 (13)	4 (11)	5 (15)	0.7
Coronary artery disease	6 (9)	5 (14)	1 (3)	0.2
Cognitive impairment	13 (19)	5 (14)	8 (24)	0.35
Stroke	2 (3)	0	2 (6)	0.2
Institutionalization	1 (1)	0	1 (3)	0.47

APACHE II - Acute Physiology and Chronic Health Evaluation II; SOFA - Sequential Organ Failure Assessment; COPD - chronic obstructive pulmonary disease. Tabagism is the only variable with significant differences between groups.

* Significant differences. Percentages may not total 100 because of rounding. Results are presented as medians and interquartile ranges or n (%).

### Changes in ventilatory management and diagnostic procedures

In 2020, a significant reduction in the use of HFNC as ventilatory support at ICU admission and a nonsignificant reduction in the use of NIV were observed compared with 2019.

**Figure 1:**
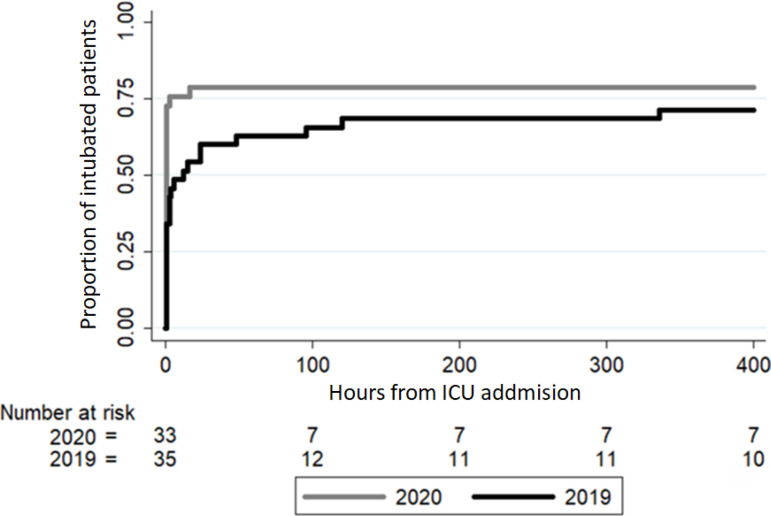
Kaplan-Meier curve of the time to intubation.

Additionally, in 2020, a significant increase in the number of patients requiring MV at ICU admission was observed. Nevertheless, the number of patients requiring MV 5 days after ICU admission was similar in both years (Table 2). The proportion of intubated patients in relation to hours in the ICU is plotted in figure 1. No significant differences in the number of days of MV and tracheostomy requirements were found.

**Table 2:** Clinical outcomes of hospitalized patients with respiratory failure in March and April of 2019 and 2020

Variables	2019	2020	p value
	(n = 35)	(n = 33)	
Clinical approach for treating respiratory failure			
Nonrebreathing reservoir mask	5 (14)	7 (21)	0.76
Noninvasive ventilation	4 (11)	2 (6)	0.54
High flow nasal cannula	15 (43)	1 (3)	< 0.001*
Invasive mechanical ventilation	11 (31)	23 (69)	0.003*
ICU complications			
Delirium	17 (49)	21 (64)	0.22
MVAP	2 (6)	3 (9)	0.66
Pressure ulcers		6 (18)	0.29
Outcomes			
Patients under MV after 5 days in the ICU	24 (69)	26 (79)	0.29
Days of mechanical ventilation	5 (3 - 8)	4 (2 - 10)	0.9
Days of ICU stay	7 (5 - 11)	11 (6 - 23)	0.26
Days of hospitalization	14.5 (8 - 26)	15 (11 - 25)	0.31
Tracheostomy	2 (6)	4 (12)	0.41
Chest tomography scan	27 (77)	7 (21)	< 0.001*
Bronchoalveolar lavage	16 (46)	3 (9)	< 0.001*
ATB change due to bronchoalveolar lavage results	5 (14)	1 (3)	0.2
Death in the ICU	12 (34)	10 (30)	0.32
Death in the general ward	1 (3)	2 (6)	0.60
Discharge from the hospital	20 (57)	19 (56)	1
Discharge to a tertiary care facility	2 (6)	2 (6)	0.99

ICU - intensive care unit; MVAP - mechanical ventilation-associated pneumonia; ATB - antibiotic.

* Significant differences. Percentages may not total 100 because of rounding. Results are presented as medians and interquartile ranges or n (%).

Proportion of intubated patients in relation to hours after intensive care unit admission in patients with respiratory failure in March and April of 2019 and 2020. Only one patient treated in 20 19 was intubated after 5 days in the intensive care unit.

ICU - intensive care unit.

A significant decrease in the number of chest CT scans and BALs performed was observed in 2020. Among 16 BALs executed in 2019, only 5 modified the antibiotic treatment, while 1 of the 3 BALs performed in 2020 changed the indicated antimicrobial therapy (Table 2).

### Intensive care unit complications and clinical outcomes

The prevalence of delirium was higher in 2020 (63%) than in 2019 (47%), without significant differences (p = 0.22). No differences were observed in either the incidence of ventilator-associated pneumonia or the report of pressure ulcers. Mortality and discharge rates were similar in both years. These results are summarized in table 2.

### Questionnaire

The questionnaire was sent to the 14 members of the ICU medical staff and 6 respiratory therapists. All 20 healthcare professionals completed the questionnaire.

Ninety percent of ICU healthcare professionals considered that a reduction in the use of NIV or HFNC occurred in 2020 in patients without COVID-19 compared with the use of these procedures in 2019. The majority of the consulted professionals believed that the cited reduction led to a higher number of patients under MV support in the ICU (Table 3).

**Table 3:** Healthcare professionals' perceptions of what changed

Questions	Answers
Which role do you perform in the intensive care unit?	
Senior intensive care unit physician	7 (35)
A fellowship trainee or critical care chief resident	7 (35)
Respiratory therapist	6 (30)
Do you consider that the use of noninvasive ventilation and high flow nasal cannulas was lower in March and April 2020 than in March and April 2019?	
Yes, the use of both high flow nasal cannulas and noninvasive ventilation was lower.	15 (75)
Yes, the use of noninvasive ventilation was lower.	1 (5)
Yes, the use of high flow nasal cannulas was lower.	2 (10)
No, I do not perceive any change in the use of noninvasive ventilation or high flow nasal cannulas.	
If your previous answer was affirmative, do you consider that the reduction in the use of noninvasive ventilation or/and high flow nasal cannulas led to a higher rate of patients requiring mechanical ventilation support?	
No	2 (10)
Yes	16 (80)
Do you consider that the performance of bronchoalveolar lavage in patients without COVID-19 was reduced during March and April 2020?	
No	4 (20)
Yes	16 (80)
If your answer to the preceding question was "yes", do you consider that the reduction in the performance of bronchoalveolar lavages had any impact on the patients' evolution?	
No	10 (50)
Yes	6 (30
Do you consider that fewer chest tomography scans were performed on patients without COVID-19 during March and April 2020?	
No	10 (50)
Yes	10 (50)
If your previous answer was affirmative, do you consider that the reduction in the number of chest tomography scans performed had any impact on the patients' evolution?	
No	5 (25)
Yes	5 (25)
Do you consider that the incidence of delirium increased in patients without COVID-19 treated in the intensive care unit during these same months?	
No	11 (55)
Yes	9 (45)
Do you consider that the incidence of pressure ulcers increased in patients without COVID-19 treated in the intensive care unit during these same months?	
No	9 (45)
Yes	11 (55)

Results expressed as n (%).

Additionally, 80% of the health care team perceived a reduction in the number of BALs in 2020, and 30% considered that this decrease had a negative impact on antibiotic therapy de-escalation. Finally, 50% replied that the number of CT scans performed was lower in the group of patients admitted in 2020 than in the group admitted in 2019. However, most of them stated that in their professional opinion, this reduction did not have any effect on the clinical evolution of patients (Table 3).

## DISCUSSION

The intensive care unit protocol for the COVID-19 pandemic based on international recommendations implied an important change in the clinical management of patients with and without COVID-19.

The use of NIV or HFNC was avoided to protect healthcare providers and to prevent the infection of other patients. The increase in the number of ventilated patients at ICU admission observed in this study might be related to this fact.^(6-8)^ Nevertheless, no changes were observed in the percentage of intubated patients 5 days after ICU admission, MV days, ICU days or hospitalization days. Based on the responses to the questionnaire, 90% of healthcare professionals noticed this reduction in the use of noninvasive ventilatory devices, and 80% considered that this reduction in the use of NIV or HFNC led to an increase in the percentage of ventilated patients.

Although NIV is associated with a lower risk of tracheal intubation,^(11)^ a low number of patients in the study population presented with chronic obstructive pulmonary disease (COPD) exacerbation or congestive heart failure, which are the two pathologies that benefit most from NIV therapy.^(12)^ The low number of patients with these pathologies and the nonsignificant reduction in the use of NIV in the 2020 group might explain why the final number of ventilated patients remained unchanged.

Secondly, a reduction in the performance of BALs in patients without COVID-19 who were hospitalized in 2020 was observed. This reduction was a consequence of the recommendation to avoid BAL in patients suspected of having COVID-19.^(13)^ Five of 16 BALs performed in 2019 affected patient evolution since the antibiotic treatment was modified, whereas 1 of 3 BALs performed in 2020 led to a change in antibiotics. These results regarding the impact of BALs are consistent with findings from the meta-analysis by Kamel et al.^(14)^ After analyzing the results of the questionnaire, 80% of healthcare professionals perceived this reduction, but only 30% considered that it had any impact on patient evolution.

Third, no significant differences in the incidence of delirium or ventilator-associated pneumonia were found. These observations are consistent with healthcare professionals’ perceptions. However, a tendency toward delirium onset was observed in the 2020 group (63% of patients) compared with the 2019 group (47% of patients). The lack of statistical significance might be explained by the low number of patients. The increase in delirium might be related to lower levels of compliance with PADIS guidelines and the A-F bundle^(15)^ in 2020 and to the application of the family visit restriction policy.

Additionally, although 55% of healthcare professionals suspected an increase in pressure ulcers, no increase was observed in the incidence of these lesions when comparing patients admitted to the ICU in 2019 with patients admitted in 2020. The misperception of this topic by the healthcare team may be related to the fact that they observed a reduction in patient mobilization.

This study has some limitations. First, the low number of analyzed patients does not allow us to arrive to a conclusion regarding ICU complications, such as delirium or pressure ulcers. Notably, we observed a tendency toward an increase in delirium and pressure ulcers in 2020 compared with 2019; however, both results were not statistically significant, although the analysis had a low power to detect this difference. Additionally, in the 2020 distribution, more women were included, whereas more men were included in 2019.

## CONCLUSION

In summary, in patients without COVID-19 who were hospitalized in the intensive care unit during 2020, the application of COVID-19 pandemic protocols reduced the usage of high-flow nasal cannula and the number of bronchoalveolar lavages and chest tomography scans, whereas it increased the number of tracheal intubations at intensive care unit admission. However, the percentage of intubated patients and the numbers of mechanical ventilation days, intensive care unit days or hospitalization days were not altered.
